# Life table study of *Sitotroga cerealella* on different cereals and its implications on the performance of the egg parasitoid (*Trichogramma chilonis*) under laboratory conditions

**DOI:** 10.1038/s41598-023-37852-0

**Published:** 2023-07-06

**Authors:** Muhammad Salim, Imdad Ullah, Ahmad Ur Rahman Saljoqi, Ayhan Gökçe, Sarir Ahmad, Mikhlid H. Almutairi, Amany A. Sayed, Lotfi Aleya, Mohamed M. Abdel-Daim, Muddaser Shah

**Affiliations:** 1 Department of Plant Protection, Faculty of Crop Protection Sciences, The University of Agriculture, P.O. Box 25120, Peshawar, Khyber Pakhtunkhwa Pakistan; 2 Department of Plant Production and Technologies, Faculty of Agricultural Sciences and Technologies, Nigde Omer Halisdemir University, Nigde, Turkey; 3 Department of Entomology, Abdul Wali Khan University, Mardan, 23200 Pakistan; 4 Department of Zoology, College of Science, King Saud University, P.O. Box 2455, Riyadh, 11451 Saudi Arabia; 5 Zoology Department, Faculty of Science, Cairo University, Giza, 12613 Egypt; 6 Chrono-Environnement Laboratory, UMR CNRS 6249, Bourgogne, Franche-Comté University, 25030 Besançon cedex, France; 7 Pharmacology Department, Faculty of Veterinary Medicine, Suez Canal University, Ismailia, 41522 Egypt; 8 Natural and Medical Sciences Research Center, University of Nizwa, P.O. Box 33, 616 Birkat Al Mauz, Nizwa, Oman; 9 Department of Botany, Abdul Wali Khan University, Mardan, 23200 Pakistan

**Keywords:** Biological techniques, Environmental biotechnology, Plant biotechnology

## Abstract

*Sitotroga cerealella* is one of the major pests of cereals in the field and storage conditions throughout the world. The main objective was to study the life tables of *S. cerealella* on wheat, maize and barley and its implications on percent parasitism of *Trichogramma chilonis*. *S. cerealella* is reared under lab conditions as its eggs are utilized for rearing *T. chilonis*. Fresh eggs of *S. cerealella* were collected and after hatching the neonate larvae of *S. cerealella* were transferred onto each host plant species for obtaining first (F1) generation (G). Seventy eggs were used for each host and each egg was used as a replicate. Daily observations were made for recording the life-table parameters of the *S. cerealella*. The data showed that the developmental time of *S. cerealella* eggs and pupae was maximum (5.68 and 7.75 days) when reared on wheat, while the maximum larval duration (19.77 days) of *S. cerealella* was recorded on barley. The maximum fecundity (290.30 ± 22.47 eggs/female) was recorded on maize, while minimum fecundity per female was recorded on barley (159.30 eggs/ female). The *S. cerealella* reared on maize had significantly higher values of finite rate of increase *(λ),* intrinsic rate of increase (*r),* and net reproductive rate (*R*_*o*_*)* (0.14 ± 0.04 day^− 1^, 1.16 ± 0.05 day^− 1^, and 136.85 ± 20.25 eggs/ female) respectively. The mean generation time (*T*) (35.18 ± 0.61 days) was higher on wheat. Likewise, the gross reproductive rate (*GRR*) and the age-stage specific reproductive values (*v*_*xj*_) of newly oviposited eggs of *S. cerealella* were recorded higher (136.85 ± 20.25; 1.160 offspring) on maize. The data regarding the efficacy of *T. chilonis* for different parameters were recorded higher on maize i.e., percent parasitism (89.00 ± 2.30%), percent adult emergence (81.60 ± 1.20%), adult longevity (3.80 ± 0.10 days) and total adult longevity (9.90 ± 0.20 days) as compared to wheat and barley. Our findings revealed that *S. cerealella* can be best reared on maize under laboratory conditions as it prefers this host as compared to wheat and barley. Therefore, assigning the most susceptible and favorite host (maize) would help us to improve *T. chilonis* mass production under laboratory conditions.

## Introduction

Cereal crops contain essential nutrients necessary for both human beings and other animals. These nutrients are needed and consumed by human beings regularly in their diet. The Food and Agriculture Organization estimated the total production of cereal crops as 2777 million tons during 2022, however the production of coarse grains used as a feed for animals, except wheat and rice reached 1330.02 million tons^1^. Global warming and other risks that have emerged in recent years show that cereal crop production and storage have critical roles in world food supply. Increasing demand for cereal crops can be met to some extent by implementing better storage conditions to reduce postharvest crop loss. Grain pests are serious threats to the stored grains and stored products in both developing and underdeveloped countries where a large portion of the stored grains and products are lost due to these storage pests^2^. The average grain losses due to the storage pests are about 12% of the total grain produced and in some cases these losses could approach 50%. An estimated 10% of stored grain losses globally are attributed to insect pest infestations^3^. This loss is caused by 70 different species of insects belonging to orders like Coleoptera and Lepidoptera. Among these insect pests, *S. cerealella* (Olivier) (Lepidoptera: Gelechiidae) is one of the most devastating primary lepidopteran pests of wheat, maize, sorghum, and rice under field and in storage conditions^4^.

The Angoumois grain moth, *S. cerealella* is one of the major pests of maize, wheat, rice, and sorghum under pre- and postharvest field and storage conditions throughout the world^5^. Damage is caused by the larvae boring into the seed and feeding on seed contents (embryo and endosperm). The silk made by the larvae on grains under high humidity imparts an unhealthy appearance, smell and intense growth of mold on grains^6^. Such damage causes substantial economic loss and reduces the viability of the seeds to germinate. Under favorable conditions, *S. cerealella* breeding may continue throughout the years in suitable stores^7,8^.

Although various control measures have been used against this destructive pest, using resistance varieties for suppression of *S. cerealella* infestation in stored products has been proposed in recent years^9^. Studying the life table parameters on resistant hosts is one of the most important tools in integrated pest management programs in stored products^10^. However, the proper use of resistant hosts in pest management techniques requires knowledge of the life table and biological parameters of pests^11^. Many scientists have used the life table theory for predicting the growth and size of populations^12,13^. Studying the age-stage, two-sex life table is a very comprehensive approach for measuring the growth, survival and reproductive potential of an insect population. This approach can faithfully represent the actual life history of an insect species since it easily depicts stage difference and incorporates both sexes. Life table studies are helpful in studying the various ecological aspects of interest in relation to insect pests and their associated natural enemies for the development of integrated control^14,15^. Traditional female-based age-specific life tables, on the other hand, neglect the male component of a population and stage structure in metamorphosing species and as a result, are fundamentally unable to accurately describe a population^16,17^. Additionally, the food quality has also been taken into consideration because it may influence the life history parameters of insects including survival rate, longevity and fecundity^18,19^.

In addition to being a serious pest of stored products, *S. cerealella* is also used as a host for mass rearing of the biological control agent, *T. chilonis.* In recent years, different cereal grains varieties have been used for large-scale production of *S. cerealella* as an alternative host for rearing of parasitoids and predators^20^. *S. cerealella* was originally used as a factitious host for the mass rearing of *Trichogramma* by Flander in 1929. Mclaren and Rye, (1983) devised an efficient method for mass rearing of *Trichogramma* wasps on *S. cerealella* eggs. The ability to rear *T. chilonis* and other biological control agents on factitious hosts like *S. cerealella* has greatly improved^21,22^. Effectiveness of bio-agents on host eggs is significantly influenced by the quality of the cereals e.g., *T. chilonis* performance was highly influenced by *Corcyra cephalonica* eggs when reared on different cereals. More adults of *T*. *chilonis* emerged on *C. cephalonica* eggs reared on maize compared to wheat and barley^23^. Although some biological parameters (development and fecundity) of *S. cerealella* on various stored products have been documented by entomologists^24,25^, a complete study of all the lifetables is not available. Therefore, the present research was designed to study the age and stage specific lifetables of *S. cerealella* and bio-efficacy of the *T. chilonis* against *S. cerealella* reared on different hosts under laboratory conditions.

## Results

The results showed that the food source affects the population parameters of *S. cerealella* (Table 1). The eggs developmental time (days) of *S. cerealella* was significantly lower when reared on maize and barely (5.45 and 5.43 days, *F* = 278.57, *DFs* = 2, 207), respectively as compared to the developmental time of *S. cerealella* eggs reared on wheat (5.68 days) (*P* = 0.0167). The total larval duration (days) of *S. cerealella* when reared on maize was recorded significantly lower (*P* = 0.0079, 17.97 days) as compared to *S. cerealella* reared on barley (19.77 days), while non-significant difference was observed when reared on maize and wheat (*P* = 0.4826, (*F* = 228.02, *DFs* = 2, 207). The data regarding the pupal duration (days) of *S. cerealella* show that significantly lower pupal duration of *S. cerealella* was recorded when reared on maize (6.57 ± 0.12 days) (*P* = 0.00, (*F* = 1142.59, *DFs* = 2, 195) as compared to wheat and barley, while non- significant difference was recorded in the pupal duration of *S. cerealella* reared on wheat (7.75 ± 0.14 days) and barley (7.42 ± 0.11 days) (*P* = 0.076). In the same way, significantly lower pre-adult survival rate of *S. cerealella* was recorded when reared on maize (30.69 ± 0.36 days) (*P* = 0.001, *F* = 0.002, *DFs* = 2, 207), while the greatest pre-adult survival rate of *S. cerealella* was recorded on barley (33.28 ± 0.33 days), followed by wheat (32.87 ± 0.39 days). The total developmental time of *S. cerealella* adults was significantly extended on maize (11.22 ± 0.30 days, *F* = 64.09, *DFs* = 2, 207) and barley (10.27 ± 0.32 days) as compared to wheat (10.90 ± 0.40 days) (*P* = 0.016). The total pre oviposition period (*TPOP*) of *S. cerealella* reared individually on barley and wheat was not significantly different (*P* = 0.5615). A significantly higher number of oviposition days of *S. cerealella* were observed when reared on maize (6.96 ± 0.360 days) (*P* = 0.00, *F* = 431.06, *DFs* = 2, 96), while no significant difference was observed in the oviposition days of *S. cerealella* reared on wheat and barley (*P* = 0.300). In the same way, significantly maximum number of eggs (fecundity/ female) of *S. cerealella* was recorded when reared on maize (290.30 ± 22.47 eggs) (*P* = 0.000, (*F* = 435.13, *DFs* = 2, 96), while no significance difference in the mean fecundity of *S. cerealella* was recorded when reared on wheat and barley (*P* = 0.082). Similarly, a significantly lower doubling time (days) of *S. cerealella* was recorded when reared on maize (6.96 ± 0.36 days, (*F* = 452.91, *DFs* = 2, 207) as compared to wheat and barley (5.37 ± 0.200 and 5.611 ± 0.227 days) respectively. The total female longevity (days) of *S. cerealella* was recorded lower on maize (42.15 ± 0.750 days, (*F* = 64.55, *DFs* = 2, 96), followed by wheat and barley with (42.84 ± 0.88, 44.72 ± 0.92 days) respectively. A non-significant difference was recorded in the female longevity of *S. cerealella* when reared on wheat and maize as compared to barley (*P* = 0.5434). Similarly, the total male longevity (days) of *S. cerealella* was recorded significantly lower on maize as compared to wheat (P = 0.006; *F* = 142.50, *DFs* = 2, 96) (41.69 ± 0.635 days), while no significant difference was observed in the mean total male longevity of *S. cerealella* reared on maize and barely (P = 0.163).

**Table 1:** Stage durations, oviposition days and fecundity (Mean ± standard error) of *Sitotroga cerealella* reared individually on wheat, barley, and maize under laboratory conditions*.*

Parameter	n*	Wheat	n	Barely	N	Maize
		x̄ ± S. E		x̄ ± S. E		x̄ ± S. E
Egg (days)	70	5.68 ± 7.423a	70	5.42 ± 0.076b	70	5.45 ± 0.059b
Larva (days)	70	18.51 ± 0.571ab	70	19.77 ± 0.45a	70	17.97 ± 0.50b
Pupa (days)	66	7.757 ± 0.148a	66	7.424 ± 0.113a	66	6.575 ± 0.125b
Preadult/immature duration (days)	66	32.88 ± 0.398a	66	33.26 ± 0.343a	66	30.70 ± 0.368b
Adult (days)	66	10.90 ± 0.409ab	66	10.27 ± 0.323b	66	11.22 ± 0.304a
TPOP (days)	33	32.87 ± 0.560a	33	33.30 ± 0.484a	33	30.69 ± 0.521b
Oviposition days	33	5.30 ± 0.303b	33	4.90 ± 0.22bc	33	6.96 ± 0.360a
Fecundity (eggs per female)	33	198.87 ± 18.89b	33	159.30 ± 12.77bc	33	290.30 ± 22.47a
Doubling time (days)	70	5.37 ± 0.200a	70	5.611 ± 0.227a	70	4.65 ± 0.154b
Female total longevity (days)	33	42.84 ± 0.88ab	33	44.21 ± 0.591a	33	42.15 ± 0.750b
Male total longevity (days)	33	44.72 ± 0.923a	33	42.90 ± 0.602ab	33	41.69 ± 0.635bc

Where **x̄** = Mean and S. E = standard error, were estimated by using the bootstrap (100000) resampling method. Figures with same letters in a row are not statistically different from each other using the bootstrap (100000) resampling technique.

*n indicates several replicates.

### Population parameters of *Sitotroga cerealella* reared on different hosts

The population parameters viz intrinsic rate of increase (*r*), finite rate of increase (*λ*), reproductive rate (*R*_*o*_), generation time (*T*) and gross reproductive rate (*GRR*) of *S. cerealella* reared individually on wheat, barley and maize are presented in Table 2. The means and standard error were estimated by using the bootstrap method. *S. cerealella* reared on maize had high values of the intrinsic rate of increase (*r*) (*F* = 531.54, *DFs* = 2, 207), finite rate of increase (*λ)* (*F* = 535.43*, DFs* = 2, 207) and net reproductive rate (*R*_*o*_) (*F* = 277.57, *DFs* = 2, 207), (0.148 ± 0.004 per day, 1.160 ± 0.005 per day and 136.85 ± 20.25 offspring) respectively as compared to *S. cerealella* reared on wheat and barley. A significant difference was recorded in the gross reproductive rate (*GRR*) of *S. cerealella* reared on maize and barley (*P* = 0.000, *F* = 306.09, *DFs* = 2, 207), while no statistical variation was recorded in the gross reproductive rate of *S. cerealella* reared on maize and wheat (*P* = 0.0865). As presented in Table 2, different hosts had a significant effect on the mean generation time (*T*) of *S. cerealella* (*F* = 253.29, *DFs* = 2, 207). The mean generation-time (*T*) was found to be lowest on maize (33.056 ± 0.642) (*P* = 0.0167) as compared to wheat (35.182 ± 0.61 days).

**Table 2:** Life table parameters (Mean ± Standard error) of *Sitotroga cerealella* reared individually on wheat, barley and maize under laboratory conditions.

Parameters	n*	Wheat	N	Barley	n	Maize
		x̄ ± S. E		x̄ ± S. E		x̄ ± S. E
The intrinsic rate of increase (*r*) per day	70	0.129 ± 0.004b	70	0.123 ± 0.004b	70	0.148 ± 0.004a
The finite rate of increase (*λ*) per day	70	1.137 ± 0.005b	70	1.131 ± 0.005b	70	1.160 ± 0.005a
The net reproductive rate (*R*_*o*_) offsprings	70	93.757 ± 14.79ab	70	75.122 ± 11.25b	70	136.857 ± 20.25a
The mean generation time (*T*) in days	70	35.182 ± 0.610a	70	34.963 ± 0.595a	70	33.056 ± 0.642b
Gross reproductive rate (*GRR*) offsprings	70	93.75 ± 14.79b	70	75.1 ± 11.25c	70	136.85 ± 20.25a

Where x̄ = Mean and S. E = standard error, were estimated by using the bootstrap (100000) resampling method. Figures with same letters in a row are not statistically different from each other using the bootstrap (100000) resampling technique.

*n indicates number of replicates.

### Survival rate of *Sitotroga cerealella* reared on different hosts

**Figure 1:**
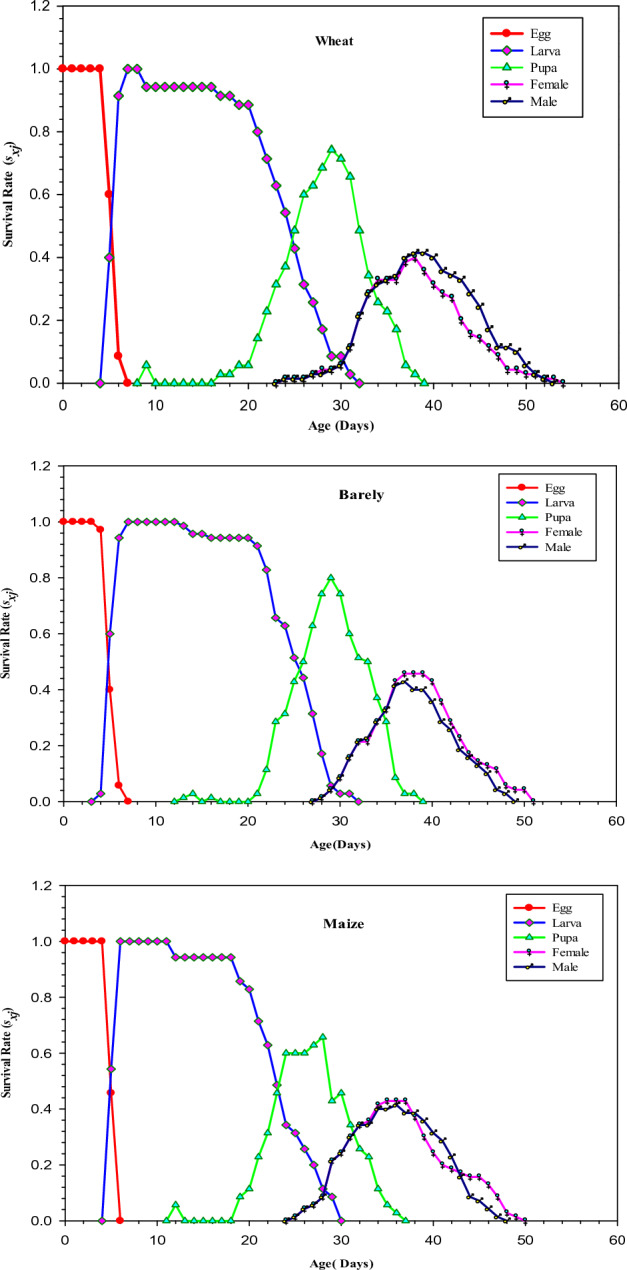
Survival rate (*s*_*xj*_) of *Sitotroga cerealella* reared individually on wheat, barley, and maize under laboratory conditions.

The age stage specific survival-rate (*s*_*xj*_) is the probability that an individual can survive to age *x* and stage *j*. The different curves in Fig. 1 showed that there was significant overlapping between the curves because of different times of development that happened among *S. cerealella* individuals. The mean (total) developmental time of *S. cerealella* eggs (Fig. 1) was significantly lower on maize and wheat (5 days) as compared when reared on barley (7 days) (*P* = 0.0167, (*F* = 64.09, *DFs* = 2, 207). Similarly, the total larval developmental duration (days) of *S. cerealella* (29 days) was noted significantly lower when reared on maize as compared to wheat and barley (*P* = 0.007, (*F* = 228.02, *DFs* = 2, 207). In the same way, less pupal developmental time of *S. cerealella* was recorded when reared on maize (37 days) as compared to barley and wheat (39 days). Similarly, the total developmental time of adult *S. cerealella* males was (48, 53 and 49 days) and for females it was (51, 54 and 51 days) when reared on maize, wheat, and barley respectively.

### Age-specific survival rate of *S. cerealella* reared on different hosts

**Figure 2:**
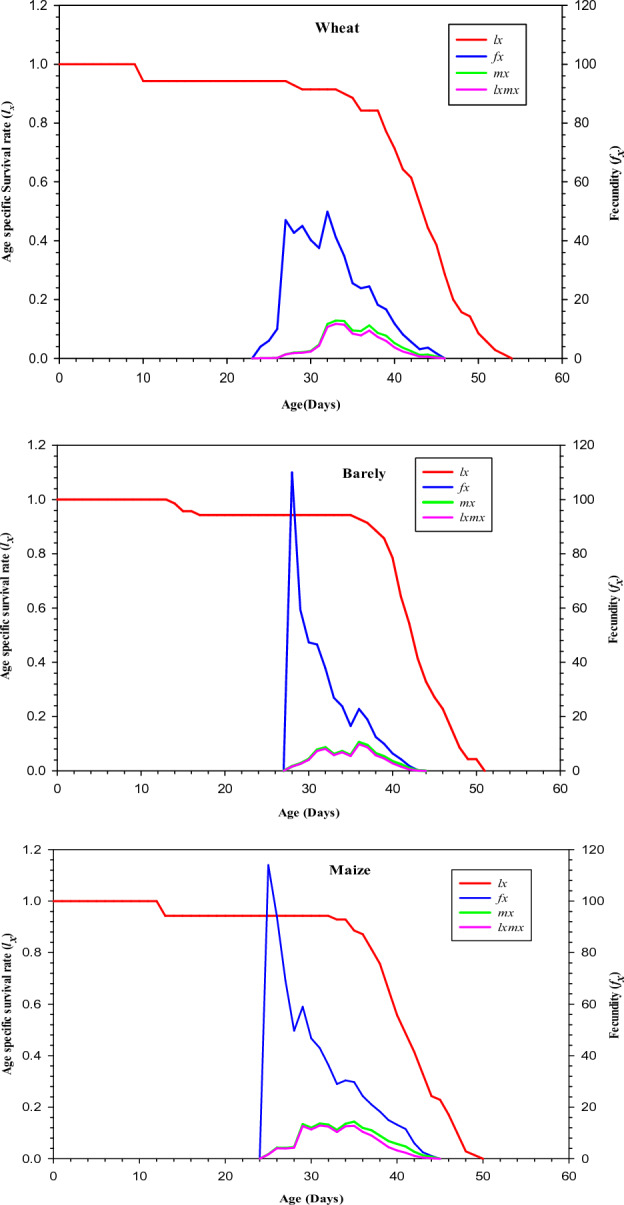
The Age-specific survival rate (*l*_*x*_), age-stage fecundity of the female stage (*f*_*x)*_ age-specific fecundity (*m*_*x*_), and age-specific maternity (*l*_*x*_*m*_*x*_) of *Sitotroga cerealella* reared on wheat, barley and maize under laboratory conditions.

The age specific survival-rate (*l*_*x*_) of *S. cerealella* dropped slowly on maize and barley as compared to wheat (Fig. 2). The value for the age-specific fecundity (*f*_*x*_) (114.00 offspring per day) was recorded on day 25 in maize which was greater than the peak value of *S. cerealella* reared on barley (110.0 per day) on day 28 and wheat (4.0 per day) on the 24th day. The curve of the age specific fecundity (*m*_*x*_) exhibit that egg-laying of *S.* *cerealella* started on day 25 and maximum egg-laying (14.38 eggs per day) of *S. cerealella* was recorded on day 25 when reared on maize, while the egg-laying of *S. cerealella* started on day 24 and maximum egg-laying (12.71 eggs per day) was recorded on day 34 when wheat was used as food source. Similarly, egg-laying of *S. cerealella* started on day 28 on barley and the maximum egg-laying (10.52 eggs per day) of *S. cerealella* was recorded on day 36. In the same way the greatest age-specific maternity (*l*_*x*_*m*_*x*_) of *S. cerealella* (12.88 offspring) was observed on maize on day 31 followed by 11.75 offspring and 9.77 offspring in wheat and barley on days 33 and 36 respectively as shown in Fig. 2.

#### Age stage-specific life expectancy

**Figure 3:**
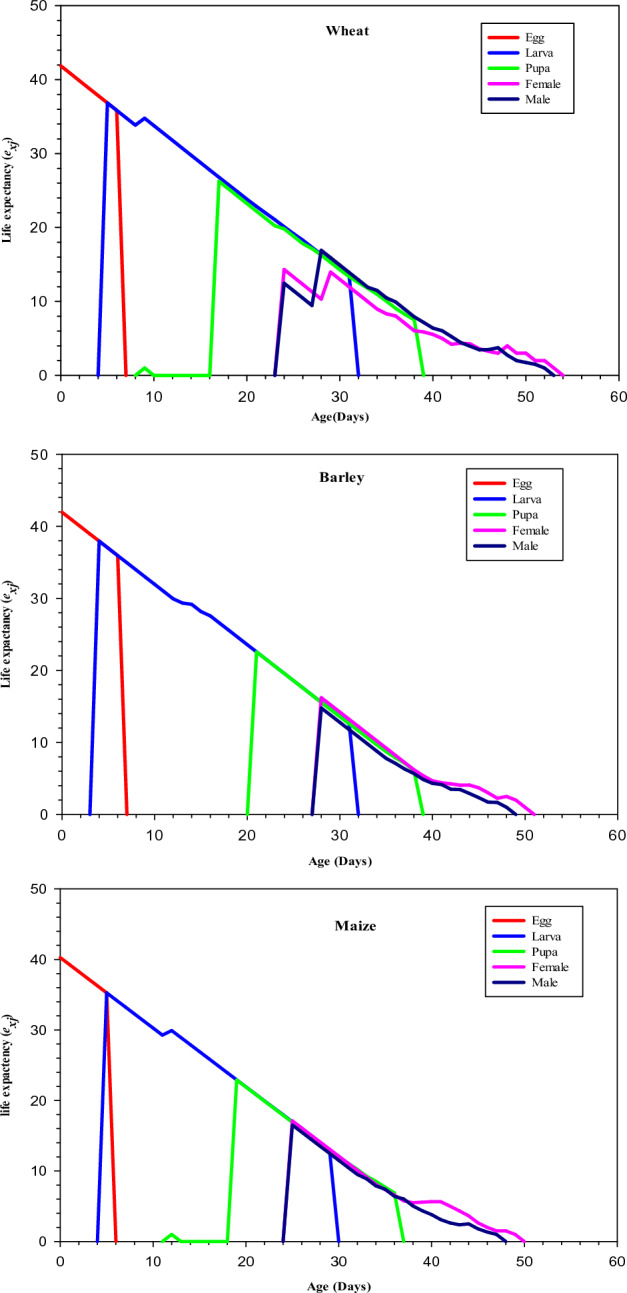
Age-stage specific life expectancy (*e*_*xj*_) of *Sitotroga cerealella* reared on wheat, barley and maize under laboratory conditions.

The age specific life-expectancy (*e*_*xj*_) of a newborn egg of *S*. *cerealella* was 41.851 and 41.942 days on maize and barley respectively which were longer than the life expectancy (*e*_*xj*_) of a newly oviposited egg of *S*. *cerealella* reared on wheat (40.271 days). As seen in Fig. 3 the life expectancy (*e*_*xj*_) of male and female *S*. *cerealella* adults was significantly high at the time of emergence (16.512 and 17.101) days respectively when reared on maize (*P* = 0.001). Similarly, the life expectancy (*e*_*xj*_) of male and female adults of *S*. *cerealella* at the time of emergence was 14.77 and 16.207 days respectively on barley while in wheat, the male and female life expectancy at the time of emergence was 12.459 and 14.318 days respectively which was lower as compared to *S*. *cerealella* reared on barley and maize (Fig. 3).

### Age stage-specific reproductive rate

**Figure 4:**
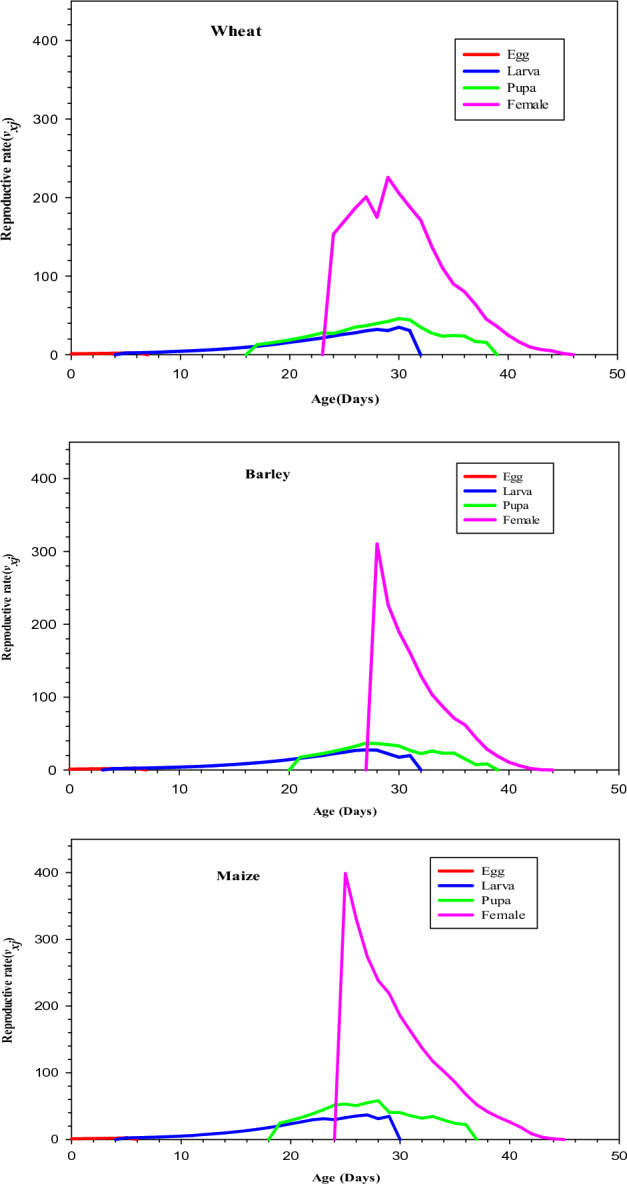
Age-stage specific reproductive rate (*v*_*xj*_) of *Sitotroga cerealella* reared on wheat, barley, and maize under laboratory conditions.

The reproductive value (*v*_*xj*_) of individuals of age *x* and Stage *j* is shown in Fig. 4. The age stage specific reproductive values (*v*_*xj*_) of a newly oviposited egg of *S. cerealella* were recorded highest (1.160 offspring per day) when reared on maize followed by the reproductive value (*v*_*xj*_) of *S. cerealella* reared on wheat (1.137 offspring per day) and barley (1.131 offspring per day) respectively. The (*v*_*xj*_) curve for the female reproductive value was significantly increased at the start of reproduction (Fig. 4). A newly emerged female showed the highest reproductive value (*v*_*xj*_) of (398.74 offspring) on day 26 when reared on maize as compared to the reproductive value (*v*_*xj*_) when reared on barley and wheat (310.39 and 153.29 offspring per day) respectively.

#### Population projection

**Figure 5:**
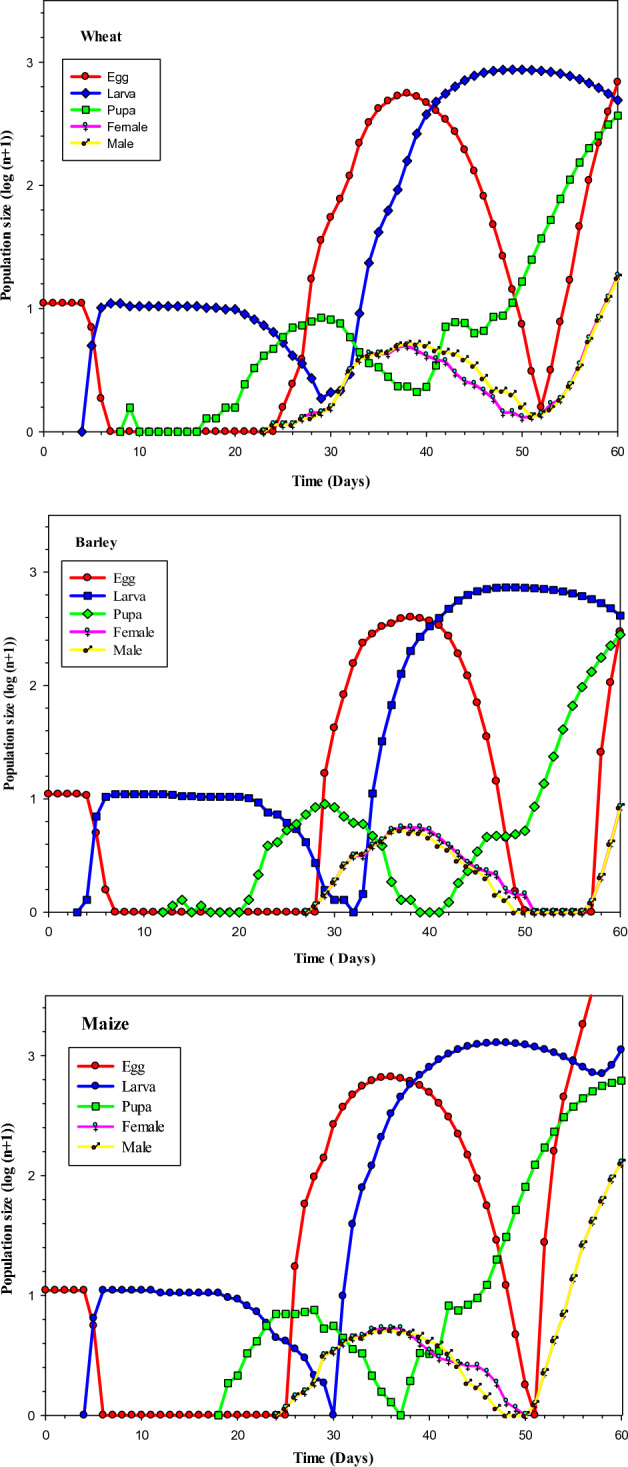
Population projection of *S. cerealella* reared on wheat, barley and maize under laboratory conditions.

The population size of the different stages of *S. cerealella* reared on maize wheat and barley simulated from an initial population of 10 eggs in each host were used to project the population growth of *S. cerealella* for 60 days duration using the TIMING-MSChart program is shown in Fig. 5. The different curves in Fig. 5 shows the trend and emergence time of different stages of *S. cerealella* reared on different hosts. According to the simulation results, the population growth of *S. cerealella* was the quickest in maize followed by wheat and slowest in barley. The *S. cerealella* population on maize reached 15,679.72 adults, on wheat reached 1,577.56 adults, and on barley reached 1,004.87 adults after 60 days (Fig. 5).

### Efficacy of *Trichogramma chilonis against Sitotroga cerealella* eggs under laboratory conditions

The mean percent parasitism of *T. chilonis* was significantly higher on eggs of *S. cerealella* reared on maize (89.00 ± 2.31%) compared with other cereals, this was followed by values for barley (77.67 ± 2.40%) and wheat (71.33 ± 1.45%) (Table 3). The maximum mean percent adults of *T. chilonis* (81.66 ± 1.20%) emerged from eggs of *S. cerealella* reared on maize followed by barley (72.00 ± 1.53%) while the minimum mean percent adult emergence of *T. chilonis* was recorded (72.00 ± 1.53%) on eggs reared on wheat. Maximum adult longevity (3.80 ± 0.12 days) and total longevity (9.96 ± 0.20 days) of *T. chilonis* were recorded on *S. cerealella* eggs reared on maize while significantly lower adult longevity (3.066 ± 0.12 days) and total longevity (7.90 ± 0.32 days) of *T. chilonis* were recorded on *S. cerealella* eggs reared on wheat.

**Table 3:** Percent parasitism, percent adult emergence, adult longevity and total adult longevity of *Trichogramma chilonis* on *S. cerealella* eggs developed on wheat, barley and maize under laboratory conditions.

Hosts	Percent parasitism (%)	Percent adult emergence (%)	Longevity (days)	Total longevity (days)
Maize	89.00 ± 2.31a	81.66 ± 1.20a	3.80 ± 0.12a	9.96 ± 0.20a
Barley	77.66 ± 2.40b	72.00 ± 1.53b	3.43 ± 0.23ab	8.50 ± 0.29b
Wheat	71.33 ± 1.45b	72.00 ± 1.53b	3.066 ± 0.12b	7.90 ± 0.32b
LSD Values	7.2648	8.1835	0.5729	0.9535

Figures with same letters in a column are not statistically different from each other using the least Significance Difference test at 5% significance level.

## Discussion

Life table studies of insects reared on different hosts can reflect the suitability of the different hosts for that specific pest species^26^. Several researchers have reported that the life table parameters of the stored grain pests could be affected by the physiochemical properties (morphology and hardness of the grain, nutritional value and availability of the food) of the stored products^27^. In the current study, the lifetable and population parameters of Angoumois grain moth, *S. cerealella* were studied individually on wheat, barley and maize. The greatest fecundity and oviposition period was recorded when *S. cerealella* was reared on maize as compared to barley and wheat. The eggs and larval developmental time (days) of *S. cerealella* was recorded lower on maize as compared to wheat and barley. Similarly, the developmental time of pupae (days) was observed lower on maize as compared to wheat and barley. Also, minimum male and female total longevity (days) was recorded when *S. cerealella* was reared on maize as compared to wheat and barley respectively. In the same way the total Pre-Ovi-Position period (*TPOP*), doubling time and pre adult survival rate (%) was recorded lower on maize and wheat as compared to barley. In previous studies, the population parameters of *S. cerealella* reared on the different hosts were reported by other researchers. A study conducted by Chi and Su reported that rapid growth of insects on a given diet suggests high host suitability^28^. Further the loss of weight, emergence of adult and gain in adult weight was observed by stored insect pests on susceptible varieties as compared to resistant varieties^24^.

The highest intrinsic rate of increase (*r*) per day for *S. cerealella* was recorded on maize and the mean generation time (*T*) of *S. cerealella* was recorded on wheat. Similarly, the highest finite-rate of increase (λ) per day, net reproductive-rate (*R*_*0*_), age specific fecundity (*f*_*x*_), age specific maternity (*l*_*x*_*m*_*x*_), life expectancy of both adults, age stage specific reproductive values (*v*_*xj*_) and Gross reproductive rate (*GRR*) of *S. cerealella* were recorded on maize. The greatest age-stage specific life expectancy (*e*_*xj*_) of newly oviposited eggs of *S. cerealella* was recorded on barley. Also, the net reproductive rate (*R*_*0*_) and gross reproductive rate (*GRR*) metrics only measure the reproductive potential of diets, not their total fitness. Therefore, care should be taken when using these two metrics (*R*_*0*_ and *GRR*) to assess if a diet is suitable for *S. cerealella*. Similar results were reported for *S. cerealella* when it was reared on maize^29^, wheat^20^ and rice^30^ and these studies show that the life table parameters of *S. cerealella* were greatly influenced by the host phenology and nutritional qualities of food sources. Another study conducted by Demissie et al., also documented that the developmental time of *S. cerealella* eggs, larvae and pupae on rice grain was 5.5 days, 25.2 days and 5.0 days respectively^31^.

Our findings on total developmental time of immature stages (33.26 days) are in complete conformity with the study of Murad and Batool, who recorded that the total developmental time of immature stages of *S. cerealella* on barley was 32.1 days^32^. Our results agree with the work of Akter et al., who reported the lifetable parameters of *S. cerealella* on different barley varieties and reported the incubation period (7.00 ± 0.00), larval period (36.80 ± 0.38), pupal period (9.48 ± 0.17) and adult longevity of both male and female (6.32 ± 0.13 and 6.74 ± 0.26) days respectively^33^.

Our results further showed that the oviposition days of *S. cerealella* on barley was (4.909 ± 0.22 eggs), with age-stage specific life expectancy (*e*_*xj*_) (41.942 days) and mean number of eggs female^− 1^ (159.30 ± 12.77 eggs) were recorded. These findings confirm the findings of Akter et al., who observed almost similar oviposition days (4.80 ± 0.25), age-stage specific life expectancy (*e*_*xj*_) ranges from 36.6–48.25 days and 161.37 ± 15.11 eggs female^− 1^ of *S. cerealella* on different cultivars of barley^33^.

The age stage specific survival rate (*s*_*xj*_) of *S. cerealella* was recorded higher on wheat as compared to maize and barely. While the survival rate of *S. cerealella* eggs, larvae and pupa reared on barley were 7, 32 and 39 days respectively. Also, the life expectancy (*e*_*xj*_) of *S. cerealella* and the age stage specific reproductive values (*v*_*xj*_) of newly oviposited egg of *S. cerealella* was high on barley as compared to wheat and maize. Similar results were also published by Rizwan et al., who studied the survival rate and other developmental parameters of *S. cerealella* on barely and recorded maximum total life expectancy of *S. cerealella* (58.459 days) on different varieties of barley^30^. Similarly, the age specific life expectancy of a newly oviposited egg of *S. cerealella* was also recorded higher on barley as compared to wheat and maize. These three hosts were further assessed for their proximate compositions (Supplementary file “Table S1”). The proximate results showed higher moisture (12.5%), crude protein (13.00%), ash content (2.20%) and crude fat (3.60%) in maize as compared to wheat and barley, while no significant different were recorded in % carbohydrate content in all the three types of grains. These results further confirm that these grain’s constituents have a direct effect on the biology of the *S. cerealella*^31^. Grains with high moisture, crude protein and ash contents are positively correlated with the progeny emerged and susceptibility index^31,32^. Similarly, grains with high crude fats have positive impact on the mean fecundity data of *S. cerealella*^10^*.* These findings support the current research finding where maximum mean number eggs laid of *S. cerealella* was recorded when reared on maize grains.

The projection of *S. cerealella* population reared on wheat, barley and maize reflects the usefulness of age-stage two-sex life tables for forecasting the population under favorable environmental conditions. According to the simulation results the population growth was the quickest on maize and it was followed by wheat and slowest on barley. The *S. cerealella* population on maize reached 15,679.72 adults, while on wheat it reached 1,577.56 adults and on barley reached 1,004.87 adults after 60 days. Predicting and anticipating the population growth of insect pests are critical in formulating the correct timing schedule for a pest management programme^34^. A computer-based program using life table data is therefore a very important tool in pest management and decision making^35^.

The mean parasitism, mean adult emergence, longevity of adult and total adult longevity of *T. chilonis* were recorded highest on *S. cerealella* eggs reared on maize. A research study conducted by Akter et al., on the lifetables of *T. chilonis* feeding on *S. cerealella* reported that *T. chilonis* parasitism ranged from 61 to 95%, with adult emergence 51 to 96.30%. They further reported adult longevity ranged from 4.2 to 7.4 days^33^. Current findings are further on par with the work of Nadeem et al., who recorded the greatest adult’s emergence (98%) of *T. chilonis* from host eggs^36^. According to evolutionary models, more females will oviposit in large hosts than in small hosts^37,38^. A similar study conducted by Khan et al., also reported the same parameters of *T. chilonis* reared on *S. cerealella* egg under free choice method and observed percent parasitism, percent adult emergence, adult longevity and total longevity of adults as 94.0 ± 0.33, 94.0 ± 0.49, 9.6 ± 0.16 and 3.6 ± 0.27) days respectively^27^. Similarly, Charnov et al. also stated that sex-ratio manipulation may be an adaptation in response to the differing impact of host size on the fitness of female versus male wasps^39^. Our findings are further in line with these studies in that large size eggs from maize produced proportionately more female wasps than smaller size eggs from wheat and barley, which produced the lowest proportion of females but still produced more females than males.

Based on present study, *S. cerealella* completed their life cycle on all tested grains but maize was the best food source among the tested grains. The simulation data results showed that the growth of the *S. cerealella* population was faster on maize than it was on wheat and barley. Also, the results show that different food sources used for rearing of host insect significantly affected the percent parasitism, adult emergence, and adult longevity of the *T. chilonis.* The current study provides valuable results since understanding the life-tables study of *S. cerealella* on various hosts will be useful in mass production of biocontrol agents. Also, utilizing a susceptible host (maize) could help researchers and farmers in improving mass production of biocontrol agents. Further study on the demographic parameters of *S.* *cerealella* can help to understand the population dynamics and to develop more effective management programs for this pest.

## Conclusions

The study results showed that *S. cerealella* completed its life cycle on all the grains however maize seems to be a better food source for mass production of this pest compared to wheat and barley. The simulation data results showed that the growth of the *S. cerealella* was faster on maize as compared to wheat and barley. Understanding the life table studies of *S. cerealella* on different hosts is useful in designing a comprehensive scheme for an IPM program of *S. cerealella*.

Additionally, *T. chilonis* parasitism rate, adult emergence, and adult longevity were affected by the food sources of host insects. Therefore, assigning the most susceptible and favorite host (maize) would help us to improve *T. chilonis* mass production. These findings will provide a concrete basis for further studies in the real environment for both *S. cerealella* and *T. chilonis*.

## Materials and methods

The study was conducted at the Department of Plant Protection, The University of Agriculture Peshawar during 2020. The insecticide-free seeds of wheat (variety Saleem 2000), barley (variety AAJ) and maize (cultivar Azam) were purchased from the local market in Peshawar. The seeds were disinfected by sterilization at 121 °C for 30 min in an autoclave. After sterilization, the seeds were air dried and then were used for the experiments.

### Rearing of the* Sitotroga cerealella*

For the rearing of *Sitotroga cerealella,* fresh eggs of *S. cerealella* were obtained from (SCRI,) Mardan during the first week of April 2020 and were maintained under laboratory conditions at 27 ± 2 °C and 65 ± 5% R.H. with 08:16 h (L: D) cycle. After hatching the neonate larvae of *S. cerealella* were transferred onto each host under laboratory conditions. These *S. cerealella* were reared on different hosts for one generation. When enough eggs (~ 1500) were produced, the life-table studies of *S. cerealella* were initiated on sterilized barley, maize and wheat.

### Life table parameters

The life table parameters of Angoumois grain moth were studied on wheat, barley, and maize under laboratory conditions at 27 ± 2 °C and 65 ± 5% R.H. with 08:16 h (L: D) cycle. After rearing for one generation, *S. cerealella* eggs were collected using a soft hand brush in Petri dishes. These eggs were then transferred into Petri dishes containing different grain hosts. A replicate consisted of a single egg and a total of 70 eggs (replicates) were used for each host as mentioned above. The cultures were maintained under the laboratory conditions at 27 ± 2 °C and 65 ± 5% R.H. with 08:16 h (L: D) cycle. A total of 70 petri dishes(replicates) were used for each diet. Daily observations were made for eggs hatching and larval duration. The pupal duration of *S. cerealella* in different hosts was confirmed by the presence of a tiny round semitransparent hole in the grains until adult emergence. Upon emergence, adults were paired and then each pair (one male and one female) was transferred into a plastic funnel (approximately 12–13 cm diameter), then covered with a lid of 75 mesh size sieve at the top face. The plastic funnel was then placed upside down over a dish containing starch for eggs data collection. The number of eggs were sieved daily through the 85-mesh size sieve and counted under a binocular magnifying instrument. The data on number of eggs were continued until the death of all individuals in the experiments.

### Population projection

The population potential and growth of *S. cerealella* reared on wheat, maize, and barley were utilized for population and growth projection of S. *cerealella* by the Chart i.e., TIMING-MS program^40,41^. For the initial population of *S. cerealella* on maize, wheat, and barley, 10 eggs in each host were used to project the population growth of *S. cerealella* for 60 days duration.

### Rearing of *Trichogramma chilonis*

When enough host eggs were available, rearing of *T. chilonis* was initiated. Sticky gum was spread uniformly on a piece of hard paper (15 × 10 cm). Then fresh eggs (300) of the *S. cerealella* (F1 generation) reared on each host were sprinkled on each sticky card pieces. These egg cards were then exposed to UV radiation for one hour to kill the larvae present inside eggs. After the radiation treatment, these eggs cards were transferred to a glass jar containing adults of *T. chilonis* in the ratio of 6:1 (female: male) for 3 h. After 3 h, the exposed egg cards were kept in separate glass jars under laboratory conditions at 25 ± 2 °C, 65 ± 5% R.H. with 08:16 h (L: D) cycle. *T. chilonis* adults upon emergence were fed on 10% honey drops solution. The emergence of *T. chilonis* adults was programmed i.e., emergence of adults from parasitized eggs which have been parasitized by keeping them at low temperatures (5 °C) during the pupal stage.

### Efficacy of *Trichogramma chilonis *against* Sitotroga cerealella* eggs under laboratory conditions

To study the efficacy of *T. chilonis* against *S. cerealella* reared on wheat, barley and maize*,* fresh eggs of *S. cerealella* (100) of F2 generation from each host were collected separately from already established cultures. Those eggs were then scattered uniformly on sticky cards (3 × 2 cm). A total of five cards each having 20 eggs/card were used for each treatment. These egg cards were then placed separately in the glass jar containing freshly mated female adult of *T. chilonis* for parasitization for 24 h. The exposed eggs were then kept in separate glass jar under laboratory conditions at 25 ± 2 °C, 65 ± 5% R.H. with 08:16 h (L: D) cycle until they turned black indicating development of the parasitoid inside. Upon turning black, the cards containing eggs were collected from the jars and were examined for percent parasitism. Percent Parasitism was calculated using formula^42^.

$${\text{\%Parasitism}} = \frac{{\text{total number parasitoid eggs}}}{{\text{total number of eggs}}} \times 100$$

### Proximate composition

Chemical analyses of the tested grains of wheat, maize and barely grains were performed using the standard official methods of analysis of Association of Official Analytical Chemists (AOAC), (2016).

### Statistical analysis

Data on eggs hatching, larval and pupal durations, adult emergence, adult longevity, preoviposition period and oviposition period were analyzed utilizing age stage, two sex life-table theory^26,28,40,43,44^. The Intrinsic rate of increase (*r*), the finite rate of increase (*λ*) and net reproductive rate (*R*_*0*_) were calculated with the help of a TWOSEX-MS Chart program. Bootstraps methods (10000 resampling) were used for the estimation of standard errors and comparison of population parameters of *S. cerealella* reared on wheat, barley and maize hosts. The data regarding the efficacy studies of *T. chilonis* were subjected to one way analysis of variances. The data on mean parasitism, mean adult emergence, adult longevity and mean (total) adult longevity were analyzed using Statistical package (Statistix 8.1) means were compared using LSD test at 5% level of probability.

### Ethical declaration

All the experiments were reviewed and approved by the Department of Plant Protection, The University of Agriculture, Peshawar ethical committee as per university set guidelines and were in accordance with the Pakistan Agricultural Pesticide Act. 1997.

## Supplementary Information

Supplementary Tables.

## Data Availability

The datasets used and/or analyzed during the current study are available from the corresponding author on reasonable request.
