# Supplementary material for: Life table study of Sitotroga cerealella on different cereals and its implications on the performance of the egg parasitoid (Trichogramma chilonis) under laboratory conditions
Source: Sci Rep. 2023 Jul 6;13:10961. doi: 10.1038/s41598-023-37852-0 (PMC10326070; doi:10.1038/s41598-023-37852-0)
Supplement: Supplementary file 1 — Supplementary Tables. [file 41598_2023_37852_MOESM1_ESM.docx]

Table S1. Chemical analysis for the nutritional compositions (%Mean± Standard error) of maize, wheat and barley grains.

| Cereals | % Moisture | % Crude Protein | % Carbohydrate | %Fat | %Ash | %Fiber |
| --- | --- | --- | --- | --- | --- | --- |
| Maize | 12.50±0.28 a | 13.00±0.05 a | 71.60±0.20 | 3.60±0.05 a | 2.20±0.05 a | 2.03±0.03 |
| Wheat | 12.16±0.16 a | 12.20±0.11 b | 71.63±0.38 | 2.20±0.05 b | 2.03±0.08 ab | 2.13±0.03 |
| Barely | 11.06±0.23 b | 8.93±0.08 c | 71.16±0.16 | 2.16±0.03 b | 1.86±0.03 b | 2.16±0.08 |
| LSD values | 0.8129 | 0.3124 | Non significant | 0.1762 | 0.2209 | Non significant |

Figures with same letters in a column are not statistically different from each other using the least Significance Difference test at 5% significance level.
